# Investigation and optimization of the piezoelectric nanocomposite ZnO/PVVH/P(VDF-TrFE) for energy harvesting applications

**DOI:** 10.1038/s41598-025-04099-w

**Published:** 2025-06-23

**Authors:** A. Sarhan, A. Hassan, M. I. Abdelhamid, T. Fahmy, A. Habib

**Affiliations:** Polymer Research Group, Physics Department, Faculty of Science, Mansoura University, Mansoura, 35516 Egypt

**Keywords:** Energy harvesting, Piezoelectric nanocomposite, P(VDF-TrFE), Electroactive β-phase, PVVH, ZnO NPs, Energy science and technology, Materials science, Nanoscience and technology, Physics

## Abstract

30/70 wt.% poly (vinyl chloride-co-vinyl acetate-co-2-hydroxypropyl acrylate) (PVVH) / poly (vinylidene fluoride-co-trifluoroethylene) P(VDF-TrFE) polymer blend (PB) are prepared and doped with various content of Zinc oxide nanoparticle (ZnO NPs) using casting technique. X-ray diffraction (XRD), Fourier transform infrared (FT-IR), Transmission electron microscopy (TEM), UV–Vis and Thermogravimetric analysis (TGA) are used for structural, optical and thermal properties investigation. XRD results revealed that the crystallinity degree of PB is enhanced from 83.8 to 92.3% upon increasing the ZnO NPs. FTIR analysis showed a shift in position of some characteristic bands, confirming the complexation between ZnO NPs and functional groups of PB. UV–Vis analysis showed that both direct and indirect energy gaps (*E*_*dg*_*/E*_*ig*_) are reduced from (4.08/2.34) for PB to (3.65/1.99) eV for 1.25 wt% ZnO/PB nanocomposite. Thermally stimulated depolarization current (TSDC) measurements demonstrated that the phase transition from ferroelectric to paraelectric phase occurred at 343 K for PB and increased to 350 K after embedding ZnO NPs. Thermal sampling (TS) technique is applied and thermodynamic parameters are estimated. Piezoelectric coefficient (d_33_) is optimized from 12.8 pC/N for PB sample to 23.7 pC/N for 1wt.% ZnO/PB nanocomposite at 6.24 × 10^5^ Pa. Our results give a prediction for new piezoelectric material design capable for various energy harvesting applications.

## Introduction

During the current decade, energy storage systems have become a subject of great interest and a major area for discussion and research as more energy is acquired from intermittently sources, due to the high fluctuations in energy requirements and supply ^1^. Energy harvesting obtained from vibration or motion of the body, like breathing or walking, using piezoelectric devices, provides an amazing way to power wearable devices. Transducers of piezoelectric energy pave the way for different energy harvesting systems that are not affected by weather and environmental conditions, unlike the other energy sources such as solar, thermal and wind energy ^2^.

Poly (vinylidene fluoride) (PVDF) and poly (Vinylidene Fluoride-Trifluoroethylene) P(VDF-TrFE) copolymer are ferroelectric polymers, due to the excellent piezoelectric activity and the ferroelectric properties show a great attracted attention, which are useful for many applications as non-volatile memories and sensors^3^. Currently, more PVDF based polymers are being improved as excellent candidates for the electric energy storage and the electromechanical actuators^4–7^. Crystalline polymorphism of PVDF is observed depending on the processing condition with five distinct phases including nonpolar α-phase (TGTG’), polar β-phase (TTTT), γ-phase (TTTGTTTG’), δ-phase (TGTG’ with two chains in different orientation) and ɛ-phase. It is known that the highly polar β-phase is the most thermodynamically stable structure and is responsible for the excellent ferroelectric and piezoelectric properties and can be obtained by electrical polarization of the α-phase under a high electric field or by mechanical stretching^8^. Besides physically modifying PVDF for obtaining the highly polar β-phase, chemically incorporating trifluoroethylene (TrFE) monomer with a certain molar content is found to be an easy and effective way for producing a highly polar β-phase^9,10^. The incorporation of the TrFE (-CF_2_-CFH-) group into the structure of PVDF plays a significant role in the phase transition behavior. The crystalline structure of PVDF modified after introducing the TrFE unit, by increasing the size of the unit cell as well as the inter-planar distance within the ferroelectric phase. The polarization is then created as a result of the attraction of most electrons to the fluorine side of the polymer chain^11^.

PVVH is an amorphous ternary polymer has chemical structure is sensitive to any variation of temperature. PVVH is widely used in sensors, membranes, corrosion inhibitors and nanogenerators^12,13^. In the literature, some articles have investigated the PVVH phenomena of relaxation as a material pure or as a polyblend with another polymer^14–16^, Also a scarcity of other researches study other physical properties, for example, optical and piezoelectric properties^17,18^.

ZnO has outstanding electrical and photonic properties as a semiconducting material and thus can be used in optoelectronics, optical sensors, piezoelectric detectors, light emitting diodes and solar cells^19^. ZnO has a wide band gap value (∼ 3.3 eV) with wurtzite structure, lower toxicity, chemically stable and cheapest material in comparison to other semiconducting materials^20,21^. Hence, the researchers are interested in fabricating the polymer-based nanocomposites with different weight fractions of inorganic nanofillers such as ZnO. These novel nanocomposites can be used as optical waveguides, lenses, light emitting diodes, optical switches, sensors, nonlinear optical devices, UV shielding material and solar cells because of the excellent absorption capability^22,23^. It is noteworthy that the properties of pure polymers could be tuned by doping with various inorganic nanofillers^24–26^. The large surface area and small size of inorganic nanoparticles play an important role for enhancing the mechanical, optical and electrical properties^27^. The scientists and researchers have reported that the electrical and optical properties of polymers are enhanced after doping with ZnO nanoparticles due to the interfacial interactions between the constituents^28^.

TSDC has become a widely used experimental technique for the investigation of slow molecular mobility, charge storage, thermal stability, charge-trapping mechanisms and relaxation process of insulating polymers ^29–31^. TSDC is a very sensitive tool to interpret the polarization process based on bulk and surface structure as well as the chemical composition. TSDC provides information on the electrical polarization and structural morphology of nanocomposite samples. The pair of charges will be connected through the non-bridging oxygen and ions from the metal oxide nanofiller, which will contribute to the local charge in the matrix of the polymer. The relaxation peak in the TSDC spectrum of the nanocomposites is contributed by orientation that depends mainly on different positions which are occupied by the dipoles or ions.

The effective improvement of the piezoelectric coefficient d_33_ for P(VDF-TrFE) takes place by introducing piezoelectric inorganic nanoparticle material, such as ZnO nanoparticles. The crystalline structure of P(VDF-TrFE) is modified after doping with ZnO nanoparticles or with other inorganic nanomaterials and consequently the polar β-phase content will be enhanced. Also, some recent fabrication methods can also enhance the polarity properties of piezoelectric flexible materials^32,33^. Since the copolymers PVDF and P(VDF-TrFE) are piezoelectric and ferroelectric polymers, they have a good piezoelectric coefficient (d_33_), which is directly proportional to the polymer crystallinity degree^34,35^.

In this study, we aim to optimize and enhance a novel piezoelectric nanocomposite structure based on PVVH, P(VDF-TrFE), and ZnO nanoparticles, which exhibits high energy harvesting efficiency. The phase transformation of the nanocomposite toward enhancing the electroactive β-phase was investigated as a novel processing approach through XRD and FTIR analysis. In addition, the direct and indirect band gap energies of the various prepared samples are calculated using UV–Vis spectra. Moreover, the thermal properties of the nanocomposite films are evaluated using TGA, TSDC and TS technique. A simple and effective approach to enhance the piezoelectric coefficient of PB doped with different ZnO NPs content is interpreted. The fabricated nanocomposite samples can pave the way for nano and micro-scale energy harvesting and optoelectronic systems.

## Experimental work

### Materials

PVVH terpolymer with molecular weight of M_W_ = 33,000 g/mol. containing 81 wt. % vinyl chloride, 4 wt. % vinyl acetate and 15 wt. % 2-hydroxy propyl acrylate is obtained from Aldrich chemical company, USA. P(VDF-TrFE) copolymer with 35 mol% TrFE and 65 mol% VDF, is provided by Solvay (Brussels, Belgium).

### Sample preparation

Pure PVVH (30 wt%) and P(VDF-TrFE) (70 wt%) are separately dissolved in DMF at 323 K for 3h and then mixed together with continuous stirring for 1h to obtain a homogeneous solution. The ZnO nanofiller is also dissolved in a mixture of DMF and acetic acid at room temperature. The solutions of polyblend and ZnO NPs are then mixed and stirred together at 323 K for 2h followed by sonication for 30 min to form ZnO/PB nanocomposites with different ZnO concentrations. Finally, all solutions are poured into glass petri dishes for 3h in an oven at approximately 373 K. The nanocomposite films are prepared with a thickness of about 30–50 μm.

### Characterization techniques

The XRD carried out for all samples using Philips PW 1390 X-ray diffractometer using a monochromator beam of Cu K_α_ radiation at λ = 1.5406 Å. In the range of 0 to 70°, the 2θ angle is scanned and the X-ray rounds are scanned at speed of 2θ = 2°/min. Fourier transformation infrared spectra are measured at temperature of 25 °C in the wavelength range of 4000–400 cm^−1^ by (IR) Spectrometer model (MATTSON 5000 FTIR). The size and dispersion of the samples are investigated by transmission electron microscope (TEM) technique by (JEOL JEM 2100) with an electron acceleration voltage of 200 kV at the national research center, Cairo, Egypt. The roughness of the samples is investigated using Image J software. UV–Vis spectra are measured for all samples in the wavelength range 190–1100 nm using UV–Vis Unicom spectrometer (Mattson, UK) at Dept. of Chem., Faculty of Sci., Mansoura Uni., Mansoura, Egypt. The samples used during UV–Vis measurements were in the form of films. TGA measurements are performed using a Shimadzu TGA-50H thermal analyzer in the range of temperature from 30 to 800 °C and flow heat rate of 15 oC /min at Chem. Dept., Faculty of Sci., Mansoura Uni., Mansoura, Egypt.

### Electrical measurements

#### Thermally stimulated depolarized current (TSDC) technique

The sample is passing through two processes known as polarization and depolarization processes. The polarization process of the samples started with heating the sample to a specific polarization temperature (T_p_), just below the glass transition temperature of the sample. At the desired temperature T_p_, a DC polarization electric field (E_p_) is applied on the sample for a certain polarization time (t_p_). Then, the sample is cooled down to room temperature in the existing external electric field. Finally, by removing the field and short-circuiting the sample for a period of time in order to eliminate rapid discharge. The next process is the depolarization process in which the sample is reheated and the resultant current is recorded using 610C Keithley electrometer.

#### Thermal sampling

The same equipment used for TSDC measurements is also used to measure TS spectra. In the TS procedure, the sample is first heated to a temperature above the polarization temperature of approximately 5 K at a constant heating rate for 10 min. The sample is then cooled to the desired polarization temperature (T_p_). Next, a polarizing electric field (E_p_) is applied for a certain polarization time (t_p_). In the presence of a polarizing field, the sample is cooled to the depolarization temperature (T_d_), which is 5 K below the polarization temperature. After the sample reaches the depolarization temperature, the field is removed and the sample is subjected to a short circuit for 10 min. Finally, the sample is cooled to room temperature. Thus, by reheating the sample at a constant rate, the current is recorded as a function of temperature, and a TS spectrum is produced for each polarization run with a polarization window (T_p_-T_d_ = 5 K).

#### Piezoelectric measurements

To perform the piezoelectric measurements, the sample is sandwiched between two metallic electrodes and the stress is applied perpendicular to the film surface. Thus, the upper film surface is supported by the load to produce piezoelectric current and measured by a Keithly 485 pm.

## Results and discussion

### XRD

**Fig. 1:**
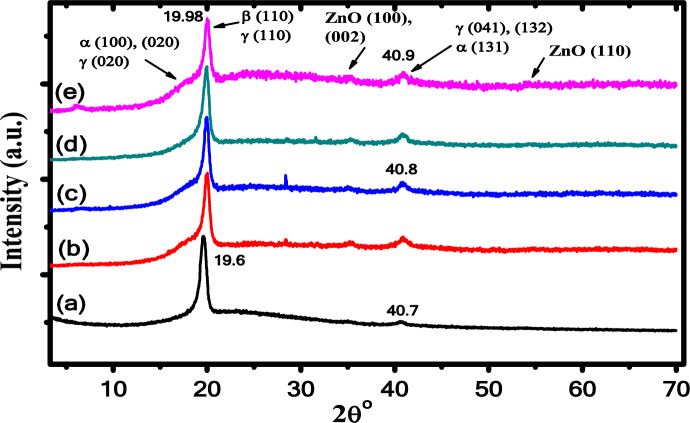
XRD pattern of (**a**) pure PB, (**b**) 0.5wt% ZnO/ PB, (**c**) 0.75wt% ZnO/PB, (**d**) 1wt% ZnO/PB and (**e**) 1.25wt% ZnO/PB nanocomposites.

X-ray diffraction is a valuable technique that provides more predictions about the sample structural change and the crystallinity information^36^. Figure 1 depicts the XRD pattern of PB samples with different concentrations of ZnO NPs. The main peak is observed at 2θ = 19.65° in the XRD pattern of PB sample corresponding to (110)/(200) plane and is related to the polar electroactive β-phase according to the card [01-072-1174]^37^, while the weak diffraction peak at 40.57° is related to (201)/(111) planes and corresponds to the β-phase crystals, confirming the presence of ferroelectric β-phase^38^, as shown in Fig. 1a. When ZnO nanoparticles are introduced to the polymer blend matrix, the position and intensity of both crystalline peaks at 19.6 and 40.8° are slightly changed with increasing the ZnO NPs content, as shown in Fig. 1b–e. These changes are due to the interaction between the polymer blend chain and ZnO NPs that leads to a decrease in the intermolecular interaction between polymer blend chains and coordination interaction between the ZnO and C–O–C group and/or C=O group of the polymer blend, which implied marked increase in the degree of crystallization^39^.

Also, a single diffraction peak is observed at 2θ = 28.3° in the ZnO/PB nanocomposite corresponds to (100) planes confirming the existence of ZnO NPs in the composite samples^40^. The crystal size (*D*), internal lattice strain (ε) and intercrystallite distance (R) of our samples are computed and listed in Table 1 using the following equations^41^:

1a $$D = \frac{0.94\,\lambda }{{\beta \,\cos \,\theta }}$$

1b $$\varepsilon = \frac{\beta }{4\,\tan \,\theta }$$

1c $$R = \frac{5\,\lambda }{{8\,\sin \,\theta }}$$

where λ is the wavelength, β is the FWHM and θ is the diffraction angle, respectively. Moreover, other structural coeffiecients such as the number of crystallites per unit area (*N*_*c*_), stacking fault (*SF*) and dislocation density (δ) are estimated with the following equations^42^:

2a $$\delta = \,\frac{1}{{\,D^{2} }}$$

2b $$SF = \beta \,\left[ {\frac{2\pi }{{45\,(\tan \,\theta )^{0.5} }}} \right]\,$$

2c $$N{}_{c} = \,\frac{t}{{\,D^{3} }}$$

where *t* is the sample thickness. The estimated structural parameters of the investigated nanocomposite samples are presented in Table 1. It is found that the crystallinity degree of PB has been enhanced after doping with ZnO NPs and increased from 83.8% of polymer blend to 92.3% for 1wt% ZnO/PB nanocomposite sample.

**Table 1:** The structural paramertes and crystallinity degree of PB and ZnO/PB nanocomposites.

Sample	*D* (nm)	*R* (nm)	*ε*_*i*_	δ (nm^-2^)	*SF*	*N*_*c*_ (nm^-2^)
Pure PB	13.51	0.562	0.015	0.0054	0.214	8.11
0.50 wt% ZnO/PB	11.68	0.558	0.017	0.0073	0.246	21.93
0.75 wt% ZnO/PB	13.11	0.554	0.015	0.0058	0.219	15.54
1.00 wt% ZnO/PB	12.72	0.555	0.016	0.0061	0.226	17.01
1.25 wt% ZnO/PB	12.53	0.554	0.016	0.0063	0.229	17.76

### FTIR spectroscopy

**Fig. 2:**
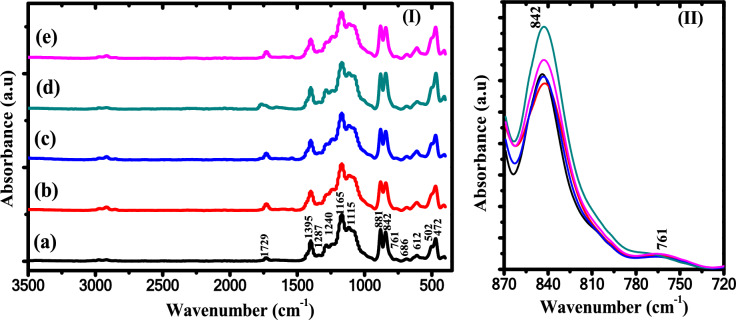
(I) FTIR spectra of (**a**) pure PB, (**b**) 0.5wt% ZnO/PB, (**c**) 0.75wt% ZnO/PB, (**d**) 1wt% ZnO/PB and (**e**) 1.25wt% ZnO/PB nanocomposites (II) The characteristic bands at 842 cm^−1^ and 761 cm^−1^.

The FTIR spectrum of PB exhibited the specific bands of both P(VDF-TrFE) and PVVH, as shown in Fig. 2I. The band at 1729 cm^-1^ is attributed to the C=O stretching vibration of carbonyl groups. The bands at 1287, 842, 502 and 472 cm^−1^ are related to all-*trans* ferroelectric β-phase of P(VDF-TrFE)^3,43^. The bands at 881 cm^−1^ and 842 cm^−1^ are ascribed to CF_2_ asymmetric stretching and to CF_2_ symmetric stretching, whereas the band at 502 cm^−1^ is assigned to CF_2_ wagging. The weak band at 761 cm^−1^ is attributed to α-phase^44^. Also, bands at 1395 cm^−1^ and 1165 cm^−1^ are ascribed to the local TrFE unit’s vibrational modes^4^. The band at 1140 cm^−1^ is attributed to C–O vibration of vinyl acetate and the band at 1115 cm^-1^ is assigned to stretching C–C bond of vinyl chloride monomer, respectively. The bands at 686 cm^−1^ and 612 cm^−1^ are assigned to the out of plane C–H deformations of C–Cl coupled with *cis* –C(H) = C(H)– stretching^18,45^. Also on the other side, the FT-IR bands of ZnO/PB nanocomposites showed that the intensity of the electroactive polar β-phase characteristic band at 842 cm^-1^ is increased as the content of ZnO NPs is increased, as shown in Fig. 2II.

**Fig. 3:**
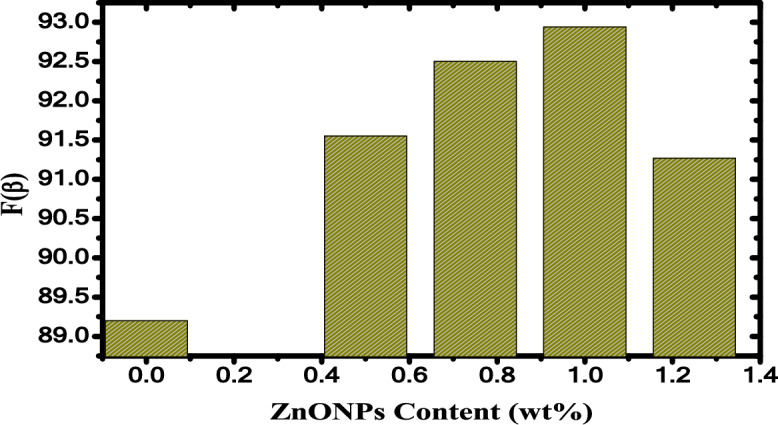
The crystallinity fraction of β-phase against ZnO NPs content.

The bands of Zn–O vibrations of ZnO are found in the range between 500 and 750 cm^−1^^25,46^. Also, it can be seen that the characteristic bands of the PB are not changed significantly with increased ZnO NPs content. The variation of the fraction *F*(*β*) of the polar crystalline β-phase in the ZnO/PB nanocomposites is estimated using the following equation^47^:

3 $$F(\beta ) = \frac{{A_{\beta } }}{{1.26\,A_{\alpha } \, + \,A_{\beta } }}$$

where *A*_*β*_ and *A*_*α*_ are the absorbance at 842 cm^-1^ and 761 cm^-1^, respectively. The high fraction of electroactive β-phase obtained at 1 wt.% of ZnO NPs and is illustrated in Fig. 3.

### TEM

**Fig. 4:**
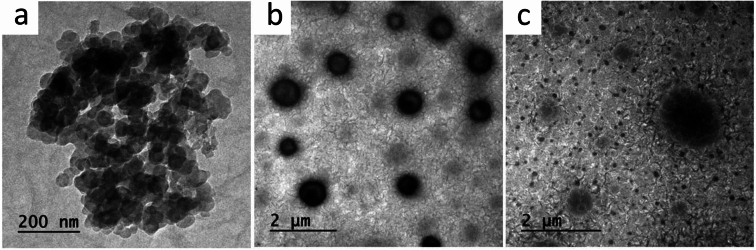
TEM patteren of (**a**) ZnO NPs, (**b**) PB and (**c**) 0.5wt.% ZnO/ PB nanocomposites.

TEM technique is carried out to investigate the morphology of ZnO/PB nanocomposites as represented in Fig. 4. Pure ZnO nanoparticles are observed with cylindrical structure of size ~ 130 nm, while PB polyblend appeared like double phase including spherical and flakelike structure of both polymers. The addition of ZnO NPs to the PB matrix produced homogeneous distribution of ZnO nanoparticles. The nano-crystals of ZnO have the structure of a cubic spinel with an average size diameter of 50 nm.

**Fig. 5:**
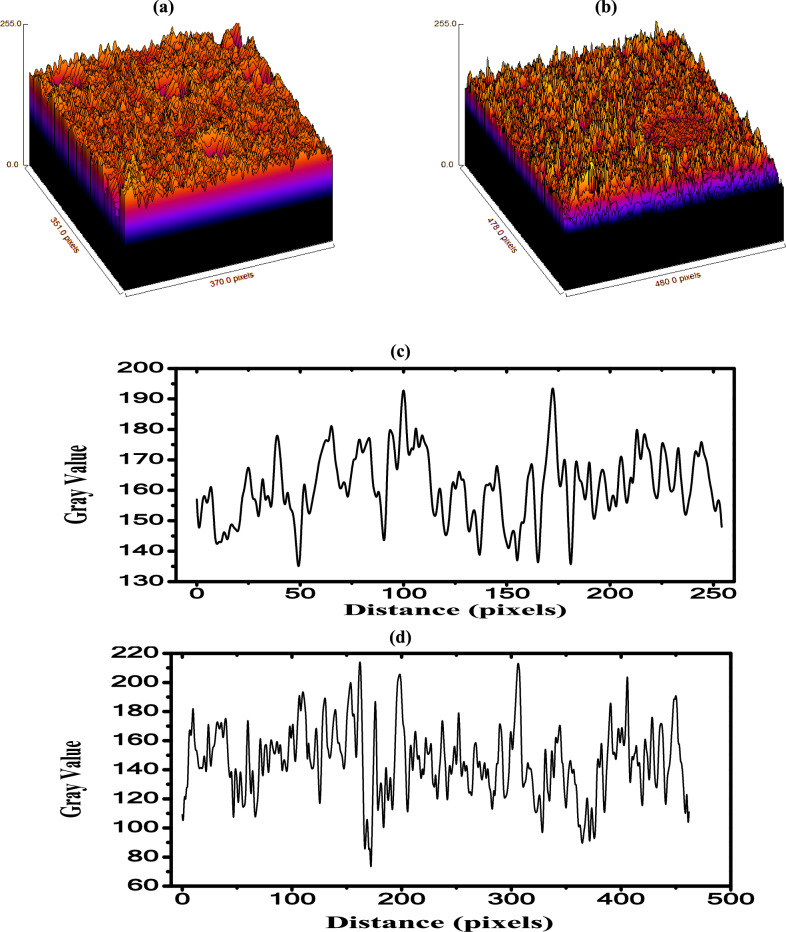
The surface roughness of (**a**) PB and (**b**) 0.5wt.% ZnO/PB nanocomposite, while the calculated roughnessparameters is presented in (**c**) for PB and (**d**) for 0.5wt.% ZnO/PB.

The surface roughness^48^ of PB and 0.5wt.% ZnO/PB nanocomposite samples have been investigated, as depicted in Fig. 5a, b. The roughness parameters are extracted from the roughness curves, as shown in Fig. 5c, d and given in Table 2. It is important to note that R_a_ refers to the average height, R_q_ is the root mean square height of the profile and R_z_ is the average maximum height. It is evident that, the values ​​of all parameters increase after the incorporation of ZnO NPs into the composite sample. This confirms that, the nanocomposite roughness is increased after presence of ZnO NPs. The porosity is also estimated and listed in Table 2.

**Table 2:** The roughness parameters of PB and PB/0.5 wt% ZnO**.**

Roughness parameters	Blend	PB/0.5 wt% ZnO
R_a_ (average roughness)	9.50	22.97
R_q_ (root mean square roughness)	11.88	29.03
R_z_ (maximum peak-to-valley height)	64.62	154.45
Porosity	1.59%	8.52%

### UV–Vis spectroscopy

**Fig. 6:**
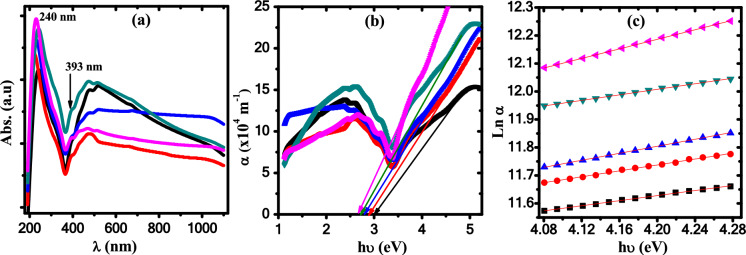
(**a**) The absorbance versus λ , (**b**) α versus hυ and (**c**) Lnα versus hυ. (black square) PB, (red circle) 0.5wt.% ZnO/PB, (blue triangle) 0.75wt.% ZnO/PB, (green reverse triangle) 1wt.% ZnO/PB and (pink leftward triangle) 1.25wt.% ZnO/PB nanocomposites.

UV–Vis spectra of the ZnO/PB nanocomposites in wavelength range of 190–1100 nm are demonstrated in Fig. 6a. The spectra of polyblend and nanocomposite samples showed an absorption peak at 240 nm and assigned to the π → π^∗^ transition originating from unsaturated bonds (C=O and C=C) of polyblend^49^. On the other hand, upon increasing the concentration of ZnO nanoparticles, the spectra of nanocomposites exhibited a shoulder at 393 nm confirming the existence of ZnO nanoparticles^50^. The absorption coefficient α (α = 2.303 *A/t*, *A* and *t* are the absorbance and sample thickness) is plotted versus photon energy for all samples as illustrated in Fig. 6b. The absorption edge (*E*_*ed*_) is estimated by Extrapolating the linear portion of the curves of Fig. 6b to intersect the x-axis at α = 0 and given in Table 3. It is clear that the absorption edge is shifted towards higher wavelength/low energy and decreased from 3.01 eV for pure polyblend to 2.67 eV for 1.25 wt.% ZnO/PB nanocomposite sample. The reduction in the absorption edge reflects the modification in the electronic structure of the polyblend matrix due to the incorporation of ZnO nanoparticles.

**Table 3:** Absorption edge and band gap energy values.

Sample	pure PB	0.5wt.% ZnO/PB	0.75wt.% ZnO/PB	1wt.% ZnO/PB	1.25wt.% ZnO/PB
*E*_*ed*_ (eV)	3.01	2.91	2.80	2.74	2.67
*E*_*dg*_ (eV)	4.08	3.97	3.87	3.77	3.65
*E*_*ig*_ (eV)	2.34	2.26	2.16	2.08	1.99
*E*_*U*_(eV)	2.22	1.92	1.61	2.04	1.17

The Urbach energy (*E*_*U*_) which indicates the band energy tails width in the forbidden gaps^51^, which can be calculated according to the following equation^52^,

4a $$\alpha \, = \,\alpha_{0} \,\,\exp \left( {{\raise0.7ex\hbox{${h\upsilon }$} \!\mathord{\left/ {\vphantom {{h\upsilon } {E_{U} }}}\right.\kern-0pt} \!\lower0.7ex\hbox{${E_{U} }$}}} \right)$$

**Fig. 7:**
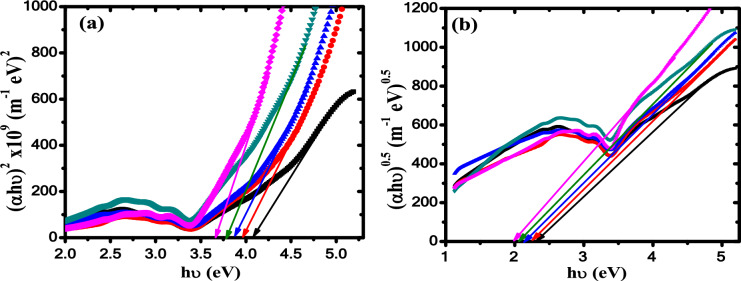
(**a**) (αhυ)^2^ vs hυ and (**b**) (αhυ)^0.5^ vs hυ (black square) PB, (red circle) 0.5wt.% ZnO/ PB, (blue triangle) 0.75wt.% ZnO/ PB, (green inverted triangle) 1wt.% ZnO/ PB and (pink leftward triangle) 1.25wt.% ZnO/ PB nanocomposites.

By plotting Ln α against hυ, as shown in Fig. 6c, and knowing the slope values of the fitted curves, the values of *E*_*U*_ are estimated and listed in Table 2. The nature of the optical transitions in ZnO/PB nanocomposites is investigated by employing Tauc’s equation as follows^53^:

4b $$\alpha h\upsilon \, = \,B\left( {E - E_{g} } \right)^{x}$$

where *E*_*g*_*, B* and *x* are optical band gap energy, an energy-independent constant and an exponent describe the transition optical nature, respectively. The identification of the transition nature based on the value of *x* which is related to allowed direct and indirect transitions and equals 1/2 and 2, respectively. The values of direct (*E*_*dg*_) and indirect (*E*_*ig*_) optical band gap energy are evaluated by extrapolating the linear portion of the curves in Fig. 7a, b to intersect the x-axis at (αhυ)^2^ and at (αhυ)^0.5^ = 0 and listed in Table 3. It is noted that both the direct and indirect are decreased from (4.08/2.34) for the pure polyblend sample to (3.65/1.99) for 1.25 wt.% ZnO/PB nanocomposite sample upon increasing content of ZnO. The reduction of the optical band gap confirms the existence of additional energy states driven by the doping of ZnO nanoparticles^32^. These results indicate that ZnO NPs tune the electronic structure of the pure polyblend due to the creation of defect levels in the band gap. The optical band gap reduction can strongly nominate our investigated nanocomposites for use as an absorbent layer in the solar cells to enhance the photovoltaic devices efficiency.

### TGA

**Fig. 8:**
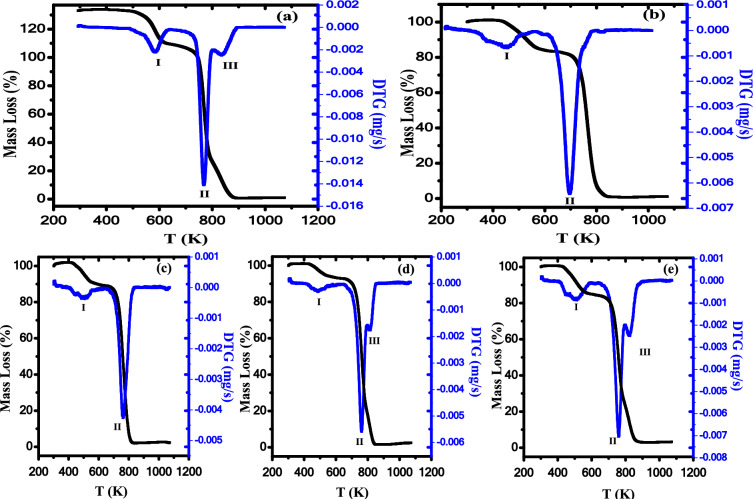
TGA curve of (**a**) PB, (**b**) 0.5wt% ZnO/PB, (**c**) 0.75wt% ZnO/PB, (**d**) 1wt% ZnO/PB and (**e**) 1.25wt% ZnO/ PB nanocomposite.

The thermogravimetric analysis (TGA) is carried out to investigate the degradation dynamics and thermal stability of the ZnO/PB nanocomposite samples. TGA/DTG curves of our investigated samples are presented in Fig. 8. It is noted that the TGA curve of PB is divided into three stages, as shown in Fig. 8a. The first stage (I) is observed in the range of temperature from 515 to 631 K with 16.14% weight loss at a temperature maximum (T_m_) of 583K. The major chemical reaction at this stage is the dichlorination reaction at the major decomposition product of HCl and low amount of Cl_2_. At this stage, acetic acid can also be removed at 523 K^12^. The second stage (II) is observed between 705 and 768 K with 59.9% weight loss at a temperature maximum of 766 K. The process of thermal decomposition of PVDF-TrFE is characterized by scissions of C-H and C-F of the polymer main-chain. The combination between fluorine and hydrogen atoms through the thermal decomposition process will promote the hydrogen fluorine (HF) formation^54^. The third stage (III) in the temperature range 805–924 K displayed a weight loss of 22.8% at *T*_m_ of 841 K, respectively. Our results showed that the second stage is the most prominent and the fastest one.

Figure 8b–e demonstrates TGA/DTG curves of ZnO/PB nanocomposite samples. It is observed that the thermal degradation of nanocomposites is achieved through three stages with lower value of maximum temperature compared to the pure polyblend sample. The values of weight loss and maximum temperature of each degradation stage are given in Table 4. It is clear that the residual mass of our samples of the nanocomposite is enhanced upon rising the content of ZnO NPs, confirming that the thermal stability of nanocomposite samples is improved due to the presence of ZnO NPs.

**Table 4:** TGA/DTG peak temperature (*T*_peak_) and the percentage of weight loss (%) for PB and their nanocomposites samples.

	Stage I		Stage II		Stage III		Residual mass %
Sample	*T*_m_ (K)	Weight (%)	*T*_m_ (K)	Weight (%)	*T*_m_ (K)	Weight (%)	
Pure PB	583	16.14	766	59.90	841	22.81	1.15
0.50 wt% ZnO/PB	506	16.00	766	81.50	–	–	2.50
0.75 wt% ZnO/PB	501	8.70	763	87.90	–	–	3.40
1.00 wt% ZnO/PB	480	7.00	758	73.57	829	16.88	2.55
1.25 wt% ZnO/PB	511	14.20	761	58.80	832	24.00	3.00

TGA is widely used to investigate the thermal degradation kinetics of polymeric materials. In this context, the mass loss of a material is recorded continuously as a function of increasing the temperature. The sample weight loss over time provided with TGA measurement is transferred to fractional conversion (g) as follows^55^:

5 $$g\, = \,\frac{{m\,_{i} - \,m_{t} }}{{m_{i} \, - \,m_{f} }}$$

where* m*_*t*_ is the mass at a certain time (isothermal analysis) over the reaction and *m*_*i*_ is the primary mass of the sample and *m*_*f*_ is the last mass at the reaction end, respectively. For material thermal degradation, a general solid-state rate of reaction is represented by fractional conversion related to time, rate constant and reaction model *f*(α). Polymer degradation kinetics is presented as follows^56^:

6 $$\frac{dg}{{dt}} = \,b\,\frac{dg}{{dT}}\,\, = k(T)\,f(g)$$

7 $$\,\,\,\,\,\,\,\frac{dg}{{dt}}\, = \,f\,\exp \,\left( { - \frac{{E_{a} }}{RT}} \right)\,\,f(g)$$

where *b*, *f*(g), *E*_*a*_, *R, k*(T) and *f* are the heating rate, differential conversion function, activation energy, universal gas constant, the rate constant and frequency factor, respectively. The frequency factor (*f*) is expressed using Eyring rate theory as follow^57^:

8 $$f\, = \,\,\frac{{\delta \,ek_{B} T_{m} }}{h}\exp \left( {\frac{\Delta S}{R}} \right)\,$$

where δ is the transmission coefficient and equals the unity for the monomolecular reaction, *e* is the Neper number (*e* = 2.7183), Δ*S* is the entropy activation and *h* is the Planck’s constant, respectively. Therefore, the rate constant can be represented as follow:

9 $$k(T)\, = \,\,\frac{{\delta \,ek_{B} T_{m} }}{h}\exp \left( {\frac{\Delta S}{R}} \right)\,\exp \left( { - \frac{{E_{a} }}{RT}} \right)\,$$

The entropy activation (ΔS) can be estimated as follow:

10 $$\Delta S\, = \,2.303\,\,R\,\log \,\,\left( {\frac{fh}{{\delta \,ek_{B} T_{m} }}\,} \right)$$

and the enthalpy activation (ΔH) can be calculated using the following equation:

11 $$\Delta H\, = \,E_{a} \, - \,RT_{m}$$

Consequently, Gibbs free energy (ΔG) can be computed as follows:

12 $$\Delta G\, = \,\Delta H\, - \,T_{m} \Delta S$$

By applying Coats-Redfern approach, the thermodynamic parameters (*f, E*_*a*_,* ΔH, ΔS, ΔG*) of pure PB and their nanocomposites can be estimated the using *n* (reaction order) = 1, according to the next equation^58^:

13 $$\log \left[ { - \frac{{\log \left( {1 - g} \right)}}{{T^{2} }}} \right]\,\, = \,\log \frac{f\,R}{{bE_{a} }}\,\, - \,\frac{{E_{a} }}{2.303RT}\, + \,\,\log \left( {1\, - \,\frac{2RT}{{E_{a}^{{}} }}} \right)\,$$

**Fig. 9:**
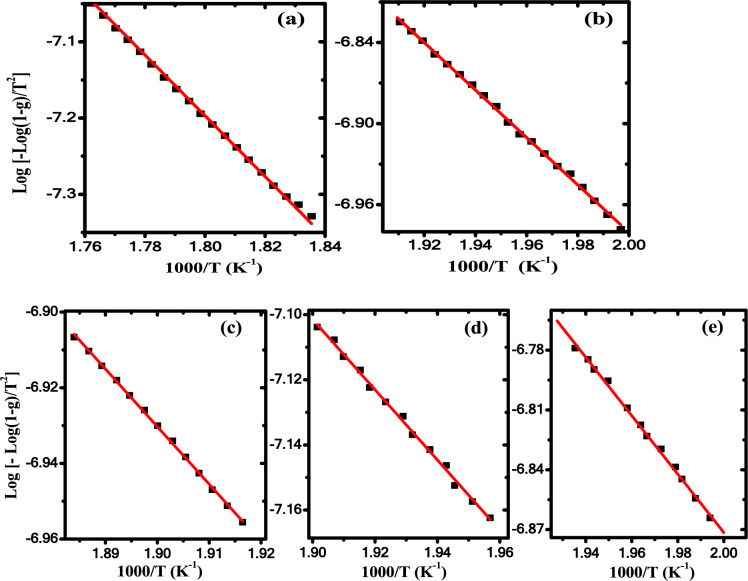
Log [−log(1 − g)/T^2^] versus 1/T (**a**) PB, (**b**) 0.5wt% ZnO/PB, (**c**) 0.75wt% ZnO/PB, (**d**) 1wt% ZnO/ PB and (**e**) 1.25wt% ZnO/PB nanocomposite in the first stage.

In case of $2RT/E_{a}$ <  < 1, the Eq. (13) will be taking the following form^55^:

14 $$\log \,\left[ { - \frac{{\log \,\left( {1 - g} \right)}}{{T^{2} }}} \right]\,\, = \,\log \frac{f\,R}{{bE_{a} }}\,\, - \,\frac{{E_{a} }}{2.303RT}\,$$

log [- log(1-g)/T^2^] is plotted versus 1/T for the polyblend and nanocomposite samples in the first stage of thermal decomposition, as shown in Fig. 9. The frequency factor *f* and *E*_*a*_ values are calculated from the intercept of log (*fR/bE*_*a*_) and slope of -*E*_*a*_*/2.303R* of the fitted curves in Fig. 9. The sample kinetic parameters in the three stages are determined and given in Table 5. The ΔS negative values as seen in Table 5 make sure that the activated complexes formation is related directly to the entropy decreasing, i.e. the complexes activated become high ordered structures compared with the beginning materials^56^.

**Table 5:** Thermodynamic parameters of ZnO/PB with different concentration of ZnO NPs .

Samples	T_m_ (K)	E (kJ)	*f* (Hz)	ΔS (kJ/mol K)	ΔH (kJ/mol)	ΔG (kJ/mol)
Stage I						
Pure PB	583	76.30	2896.707	−0.189	71.461	181.93
0.5 wt% ZnO/ PB	527	33.61	0.457407	−0.262	29.230	167.66
0.75wt % ZnO/ PB	505	29.13	0.106628	−0.274	24.936	163.50
1 wt % ZnO/ PB	475	20.66	0.00736	−0.296	16.714	157.60
1.25wt % ZnO/ PB	511	28.05	0.128864	−0.272	23.810	163.21
Stage II						
Pure PB	766	239.16	8.85E + 13	0.011	232.79	224.16
0.5 wt% ZnO/ PB	766	181.33	1.12E + 10	−0.063	174.96	223.51
0.75wt % ZnO/ PB	756	158.21	2.59E + 08	−0.094	151.93	223.51
1 wt % ZnO/ PB	762	198.33	1.65E + 11	−0.040	191.99	223.22
1.25wt % ZnO/ PB	761	125.89	1,216,791	−0.139	119.56	225.54
Stage III						
Pure PB	841	95.31	4883.482	−0.185	88.321	244.03
0.5 wt% ZnO/ PB	–	–	–	–	–	–
0.75wt % ZnO/ PB	–	–	–	–	–	–
1 wt % ZnO/ PB	825	237.4	2.09E + 13	−0.001	230.54	231.13
1.25wt % ZnO/ PB	828	117.03	166,271.7	−0.155	110.15	239.16

**Fig. 10:**
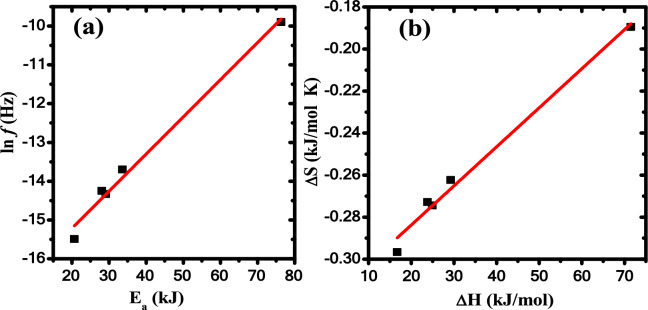
a) ln* f* versus *E*_*a*_ and b) ΔS versus ΔH in the first degradation stage of ZnO/ PB nanocomposites.

As clear from Table 5, there is a relationship between activation energy (*E*_*a*_) and frequency (*f*) i.e., a large value of *f* is accompanied by a large value of *E*_*a*_ and a vice versa, such behavior often is called the phenomenon of compensation ^59^, as shown in Fig. 10a. Moreover, a linear relation is found between (ΔS) and (ΔH) as illustrated in Fig. 10b. The ΔS and ΔH linear relation confirms the presence of the compensation phenomenon in PB and their nanocomposites. Such behavior between ΔS and ΔH is called enthalpy-entropy compensation (EEC) effect. This compensation effect explains the enthalpy variation arising from temperature changes during the molecular splitting of the polymeric material when the degradation process is compensated with the variation in the activation of enthalpy variation^60^. EEC effect has been reported previously using TGA/DTG analysis or thermally stimulated depolarization current technique for many polymers^12,14^.

### TSDC

It is well known that global TSDC spectra depend mainly on the materials under investigation, so either a single peak with specific characteristics or a broad peak containing many overlapped peaks can be obtained^61^. The spectrum of TSDC obtained from the reorientation process of dipoles in polymer matrix and impurities migration or the space charge polarization. In our previous work we investigated the TSDC spectrum of both PVDF-TrFE and PVVH separately in more detail^18^.

**Fig. 11:**
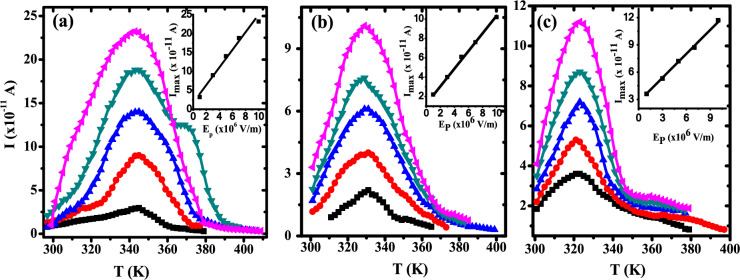
TSDC of (**a**) pure PB, (**b**) 0.5wt.% ZnO/ PB and (**c**) 1wt.% ZnO/ PB at different poling field (E_p_). (black square) 1 × 10^6^ V/m, (red circle) 3 × 10^6^ V/m, (blue triangle) 5 × 10^6^ V/m, (green inverted triangle) 7 × 10^6^ V/m, (pink leftward triangle)1 × 10^7^ V/m.

Figure 11 represents TSDC global spectra of PB and their nanocomposite samples. TSDC spectrum of polyblend exhibited a main relaxation peak that centered at 343 K and a shoulder in the high temperature region ranged from 380 to 400 K, as shown in Fig. 11a. The relaxation peak is attributed to the ferroelectric-paraelectric phase transition, while the shoulder is due to the de-trapped space charges which are injected during the process of polarization in the paraelectric phase^62^. This relaxation is called space charge relaxation and termed as ρ-relaxation. Figure 11b, c displays the global TSDC of ZnO/PB nanocomposite samples with different contents of ZnO as representative samples of nanocomposites. It is noticed that global TSDC of nanocomposite samples has the same relaxation peaks as observed in the PB, but with a shift in the peak position towards lower temperature with the concentration of ZnO nanoparticles increased. The variation of the maximum current (I_m_) of global TSDC spectra against the polarizing electric field (E_p_) is used to differentiate between the origins of TSDC peaks. The linear behavior between I_m_ and E_p_ of the relaxation peak for all samples, as shown in the inset of Fig. 11, and the fixed position of the relaxation peak indicate that the most relaxation processes contributions are due to the permanent dipoles, i.e., dipolar relaxation^63^.

### Thermal sampling

**Fig. 12:**
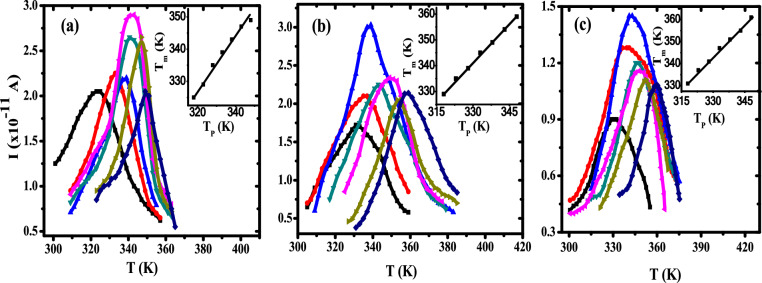
TS plots of (**a**) pure PB, (**b**) 0.5wt.% ZnO/ PB and (**c**) 1wt.% ZnO/ PB at different polarizing temperatures (T_p_). (black square) 318K, (red circle) 323K, (blue triangle) 328K, (green inverted triangle) 333K, (pink leftward triangle) 338K, (yellow rightward triangle) 343 K and (blue diamond) 348K.

The technique of thermal sampling (TS) is carried out to resolve the TSDC spectra of the investigated samples to its elementary peaks, as illustrated in Fig. 12.

The broadness of some TS relaxation peaks obtained corresponds to interactions of different relaxation modes and can be explained in terms of the distribution of relaxation times^64–66^. It is found that the obtained isolated primary peaks shifted to higher temperature with increasing polarization temperature over a wide range indicating that a broad distribution of relaxation time has taken place. It is observed that the maximum temperature (T_m_) of each TS peak varies linearly with the polarization temperature (T_p_), as shown in the inset of Fig. 12, with a slope approaching unity, indicating the presence of a continuous distribution also in the activation energy and relaxation time^67^. The molecular parameters of each TS relaxation peak are evaluated and summarized in Table 6.

**Table 6:** The molecular parameters of PB and ZnO/PB nanocomposite samples from TS spectra.

Sample	T_P_ (K)	T _max_ (K)	E_a_ (eV)	_τo_ (s)
Pure PB	318	325	0.148	6.13768
	323	329	0.386	0.00057
	328	335	0.306	0.01536
	333	339	0.563	1.5E-06
	338	343	0.569	1.5E-06
	343	347	0.584	1.2E-06
	348	349	0.402	0.00081
0.5 wt% ZnO/PB	318	329	0.222	0.32467
	323	335	0.227	0.32422
	328	339	0.446	0.0001
	333	345	0.337	0.00705
	338	349	0.534	7.4E-06
	343	354	0.613	6.4E-07
	348	359	0.764	5.4E-09
0.75 wt% ZnO/PB	318	333	0.408	0.00031
	323	339	0.276	0.05465
	328	342	0.268	0.08169
	333	349	0.652	1.2E-07
	338	353	0.275	0.08998
	343	357	0.434	0.00037
	348	363	0.481	9.7E-05
1 wt% ZnO/PB	318	331	0.197	0.93752
	323	337	0.272	0.06032
	328	341	0.335	0.00651
	333	347	0.219	0.62334
	338	351	0.2392	0.32622
	343	355	0.361	0.00447
	348	361	0.361	0.00563
1.25 wt% ZnO/PB	318	327	0.303	0.01277
	323	331	0.226	0.2979
	328	337	0.136	13.2393
	333	341	0.281	0.04945
	338	347	0.507	1.8E-05
	343	351	0.393	0.00121
	348	355	0.656	1.6E-07

According to Eyring rate theory the relaxation time (τ) of each elementary peak in TS spectrum can be expressed in terms of Gibbs free energy (ΔG) as follows^68^:

15 $$\tau = \frac{h}{{k_{B} T}}\,\exp \left( {{\raise0.7ex\hbox{${\Delta G}$} \!\mathord{\left/ {\vphantom {{\Delta G} {k_{B} T}}}\right.\kern-0pt} \!\lower0.7ex\hbox{${k_{B} T}$}}} \right)$$

From Eq. 12, Eq. 15 will take the following form as follows:

16 $$\tau = \frac{h}{{k_{B} T}}\,\exp \left( {{\raise0.7ex\hbox{${\Delta H\, - \,T\,\Delta S}$} \!\mathord{\left/ {\vphantom {{\Delta H\, - \,T\,\Delta S} {k_{B} T}}}\right.\kern-0pt} \!\lower0.7ex\hbox{${k_{B} T}$}}} \right)$$

17 $$\ln \,\left( {\frac{{\tau Tk_{B} }}{h}} \right) = \,\,\frac{\Delta H}{{k_{B} T}}\,\, - \,\frac{\Delta S}{{k_{B} }}\,$$

**Fig. 13:**
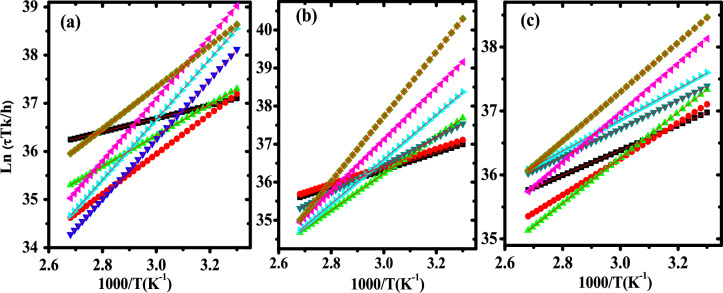
Ln (τTk_B_/h) against 1000/T of (**a**) pure PB, (**b**) 0.5wt.% ZnO/PB and (**c**) 1wt.% ZnO/PB at different polarizing temperatures. (black square) 318K, (red circle) 323K, (blue triangle) 328 K, (green inverted triangle) 333 K, (pink leftward triangle) 338K, (yellow rightward triangle) 343 K and (blue diamond) 348K.

Figure 13 displays the relaxation map (RM) of pure PB and representative samples of nanocomposites by plotting ln (τ*Tk*_*B*_*/h*) against 1/T. The compensation phenomenon of ZnO/PB nanocomposites has been verified by intersection of some straight lines, as shown in Fig. 13. The intersection point is called the compensation point and each compensation point is characterized by two characteristic parameters, the temperature of compensation (T_c_) and the relaxation time of compensation (τ_c_). Values of (T_c_) and (τ_c_) are estimated and summarized in Table 7. It is found that the polyblend and its nanocomposites are characterized by two compensation points, indicating that these materials contain a certain set of molecules that have values such as the free energy change at these points^69^.

**Table 7:** The parameters of compensation, thermal expansion coefficient and degree of disorder of PB blend and ZnO/PB nanocomposite samples.

Sample	T_c_ (K)	τ_c_(s)	DOD (cal deg^−1^)	β`(K^−1^) × 10^–5^	∆β`(K^−1^) × 10^–5^
Pure PB	347.22	558.94	28.42	72.0	32.0
	360.75	399.34		69.3	30.8
0.5 wt% ZnO/PB	361.01	498.24	27.94	69.3	30.8
	340.71	740.23		73.4	32.6
0.75wt% ZnO/PB	343.64	1054.06	27.37	72.8	32.3
	366.03	600.21		68.3	30.4
1 wt% ZnO/ PB	373.13	577.13	27.48	67.0	29.8
	348.43	950.09		71.8	31.9
1.25wt% ZnO/PB	364.16	519.25	27.79	68.7	30.5
	340.71	817.26		73.4	32.6

Density of the disorder (DOD) of the ZnO/PB nanocomposites is computed by the compensation point coordinates (T_c_&τ_c_) using the following eqn. and listed in Table 7^70^:

18 $$DOD = \,\,100 - \,2\,\left[ {\ln \,\left( {T_{c} \tau_{C} } \right)\, + \,23.76} \right]$$

**Fig. 14:**
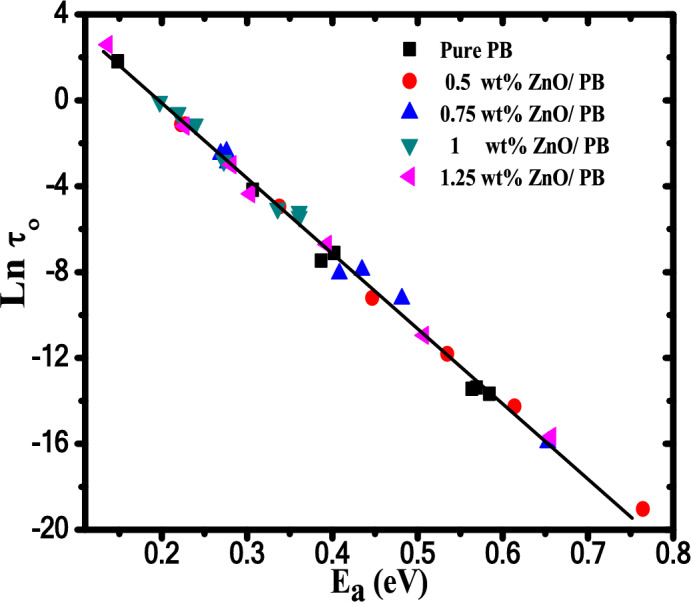
Lnτ_o_ versus E_a_ for pure PB and other nanocomposite samples from TS spectra analysis.

Values of DOD reflect the compatibility degree between the different phases of polymer blends and polymer nanocomposites^71,72^. It is found that the addition of ZnO NPs decreases the value of DOD of ZnO/PB nanocomposites. Values of DOD for our inspected polymer nanocomposite are in between small periods range from 27.37 to 28.42 (Cal deg^-1^), while normally the values of DOD change from 30 to70 for the amorphous polymers^72^. The effect of compensation can be recorded by a linear relationship between the activation energy (E_a_) and the pre-exponential factor (τ_o_), as shown in Fig. 14.

Thermal volume expansion coefficient (β` = 1/4T_c_) and the coefficient of thermal expansion difference (Δβ`=1/9T_C_) above and below the temperature of glass transition (T_g_) of our investigated nanocomposites are evaluated by the T_c_ values and given in Table 7^73^.

### Piezoelectric effect

**Fig. 15:**
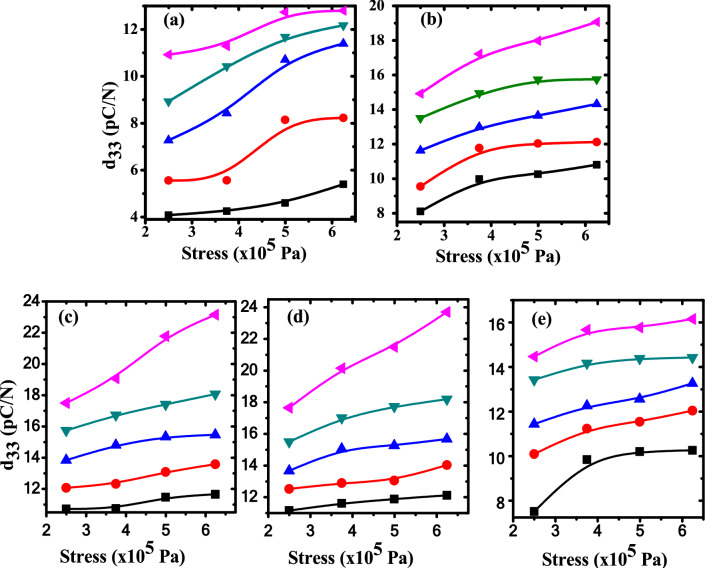
The piezoelectric coefficient (d_33_) against the stress at different temperatures for (**a**) pure PB, (**b**) 0.5wt.% ZnO/PB, (**c**) 0.75wt.% ZnO/PB, (**d**) 1wt.% ZnO/PB and (**e**) 1.25wt.% ) ZnO/PB nanocomposites, the samples are poled at E_p_ = 1 × 10^7^ V/m, T_p_ = 353 K for t_p_ = 20 min. (black square) T_m_ = 313 K, (red circle) T_m_ = 323 K, (blue triangle) T_m_ = 333 K, (green downward triangle) T_m_ = 343 K, (pink leftward triangle) T_m_ = 353 K.

Figure 15 displays the piezoelectric coefficient (d_33_) dependence on the applied stress at various measuring temperatures for pure PB sample and ZnO/PB nanocomposites. The piezoelectric coefficient d_33_ is calculated from the following relation^33^:

19 $${d}_{33}=\frac{{Q}_{3}}{A} \frac{{A}^{o}}{F}$$

where Q_3_ is the charge in z-direction of the sample, A is the electrode area, A^o^ is the area of applied load and F is the force in z-direction.

The calculated values of piezoelectric coefficient d_33_ of ZnO/PB nanocomposites are dependent on the applied stress and measuring temperature T_m_. It is observed that the piezoelectric coefficient d_33_ is increased nonlinearly upon increasing the applied stress. According to the dimensional effect model, the distance of the inter-chain can be easily changed by applying the external stress. As a result of that, the external applied stress affects strongly on the d_33_ coefficient^74^. Also, more dipoles within the samples will be rotated upward as a result of applying pressure to the samples, resulting in an increase in the polarization of the sample dipole, ΔP, which is also an effective electric field produced in the sample plane. This interaction electromechanically is actually producing a high piezoelectric effect^75^. It is also found that d_33_ values ​​increased with increasing measuring temperatures (T_m_) for all samples. As the temperature increases, the molecular chain motion will increase, making the dipole moments align better with the applied electric field, thus enhancing the piezoelectricity^76^.

**Fig. 16:**
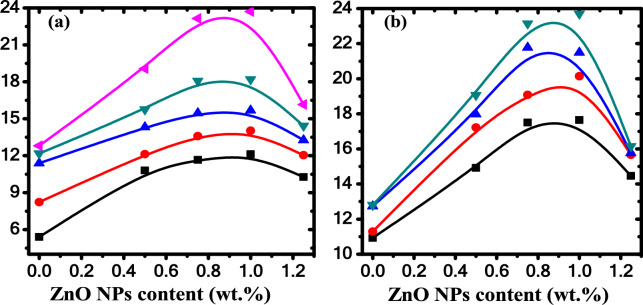
(**a**) The piezoelectric coefficient (d_33_) against ZnO content at different measuring temperature T_m_ for different samples that poled with E_p_ = 1 × 10^7^ V/m at T_p_ = 353K for t_p_ = 20 min and the applied stress is 6.24 × 10^5^ Pa. (black square) T_m_ = 313 K, (red circle) T_m_ = 323 K, (blue triangle) T_m_ = 333 K, (green inverted triangle) T_m_ = 343 K, (pink leftward triangle) T_m_ = 353 K, (**b**) the piezoelectric coefficient (d_33_) against ZnO content at measuring temperature T_m_ = 353K with different applied stress. (black square) 2.49 × 10^5^ Pa, (pink circle) 3.74 × 10^5^ Pa, (blue triangle) 4.99 × 10^5^ Pa, (green inverted triangle) 6.24 × 10^5^ Pa.

Figure 16a depicts the dependence of piezoelectric coefficient on the ZnO NPs concentration at various measuring temperatures T_m_ with fixed applied stress at 6.24 × 10^5^ Pa. It is observed that the measured value of d_33_ increases non-linearly to a maximum value at 1 wt.% ZnO nanoparticle and then decreases. This may be due to the saturation of the ferroelectric domains at this concentration. The defects increased in the polymeric chain of the PB blend after embedding ZnO NPs will increase the polarity of the samples, which magnify the piezoelectric response. Figure 16b presents the variation of piezoelectric coefficient (d_33_) versus the ZnO NPs content at various applied stress and a constant measuring temperature at 353 K.

It is found that the piezoelectric coefficient d_33_ of PB is enhanced from 12.8 pC/N to be 23.74 pC/N for PB/1 wt.% ZnO nanocomposite sample at 6.24 × 10^5^ Pa. It can be concluded that the increasing of ZnO content results in an increasing of the electroactive β-phase in the nanocomposite samples and thus enhanced the piezoelectric response.

## Conclusion

XRD and FT-IR measurements presented an improvement in the degree of crystallinity and electroactive β-phase of PB upon increasing ZnO NPs. The reduction in both (*E*_*dg*_*/E*_*ig*_) form (4.08/2.34) for pure PB to (3.65/1.99) eV for 1.25 wt% ZnO/PB nanocomposite is attributed to the creation of new localized states in the energy gap region. Global TSDC spectra of pure PB and ZnO/PB nanocomposites showed two different relaxation modes. The first one interpreted the ferroelectric-paraelectric phase transition and was named as a dipolar relaxation whereas, the second took place in the temperature range ~ 380–400 K and named as a space charge relaxation. These global spectra are decomposed into its elementary peaks by applying TS technique and hence, using the Eyring rate theory, the thermodynamics coefficients are estimated. Our results demonstrate that the electroactive β-phase of pure PB is enhanced after the introduction of ZnO nanoparticles, thereby improving the piezoelectric coefficient of nanocomposite samples. Therefore, we can conclude that ZnO/PB nanocomposites offer a novel structure applicable to a variety of flexible electronic and electrical applications, such as nanogenerators, sensors, power sources and energy storage.

## Data Availability

The datasets used and/or analyzed during the current study are available from the corresponding author on reasonable request.
